# A One-Step Approach for a Durable and Highly Hydrophobic Coating for Flax Fabrics for Self-Cleaning Application

**DOI:** 10.3390/molecules29040829

**Published:** 2024-02-13

**Authors:** Antoine Ishak, Rodolphe Sonnier, Belkacem Otazaghine, Claire Longuet

**Affiliations:** PCH, IMT–Mines Alès, 6, Avenue de Clavières, 30100 Alès, France; antoine.ishak@mines-ales.fr (A.I.); rodolphe.sonnier@mines-ales.fr (R.S.); claire.longuet@mines-ales.fr (C.L.)

**Keywords:** flax fabric, hydrophobisation, self-cleaning, PDMS, hydrosilylation, coating

## Abstract

Highly hydrophobic flax fabrics with durable properties were prepared using the “dip-coating” method for self-cleaning application. Flax fabrics were coated with a polysiloxane coating via a hydrosilylation reaction with a Karstedt catalyst at room temperature. The coated fabrics displayed highly and durable hydrophobic properties (contact angle and sliding angle of about 145° and 23°, respectively) with good self-cleaning ability for certain pollutants and excellent durability. Moreover, the influence of the coating process on the mechanical properties of fabrics was investigated. A decrease in E modulus and an increase in tensile stress at maximum force and elongation at maximum force has been observed. Furthermore, this influence of the coating process can be easily controlled by adjusting the proportion of curing agent in the treatment solution.

## 1. Introduction

Recently, hydrophobic fabrics have attracted great interest, especially in the applied research field, due to their different applications, such as self-cleaning [1,2,3,4,5,6,7,8,9,10], water/oil separation [11,12,13,14] and antibacterial [15,16] and antifouling materials [17,18]. Several methods have been suggested by researchers for preparing hydrophobic natural fabrics, such as dip-coating [19,20,21,22,23,24], spray coating [25,26], self-assembly [27,28,29] and grafting [30,31,32]. Among these methods, dip-coating is the most used due to some advantages such as low cost [24] and simplicity compared to other methods, because it does not require particular equipment, and the coating film can be successfully prepared at simple laboratory conditions. Furthermore, dip-coating is suitable for the continuous industrial production of fibrous products such as filaments or fabrics [33], besides giving good durability for the prepared fabrics [34]. Generally, this method is carried out by dipping the fabric in the coating solution, followed by drying and curing. For hydrophobic applications, the coating solution contains hydrophobic agents to decrease the surface energy of the coating layer [35]. Silicone compounds such as polydimethylsiloxane PDMS have been used in many protective coating systems, especially for hydrophobic applications. PDMS films display very stable chemical bonds on their surfaces, and their silica (-Si-O-) network is also able to be incorporated with another function group to increase the mechanical properties [36]. Many researchers have used PDMS in the design and production of hydrophobic fabrics [37,38,39,40]. For example, Talebizadehsardari et al. [41] have prepared a hydrophobic cotton fabric via the dip-coating method using hydroxyl-terminated PDMS with tetraethyl orthosilicate (TEOS) as a curing agent, dibutyltin dilaurate (DBTDL) as a catalyst and THF as a solvent. The fabrics were coated by immersion into the coating solution for 30 min, followed by drying at 80 °C for 30 min. The catalytic crosslinking reaction allows a PDMS coating to be produced on the surface of cotton fabrics. The water contact angle for the coated fabric was 123°. But it increased up to 156° when the authors added silica nanoparticles to the coating solution.

Indeed, it is possible to disperse micro/nano-particles such as TiO_2_, SiO_2_ and ZnO in the coating solution in order to integrate these particles into the coating layer in order to increase the roughness of the surface, which allows superhydrophobic fabrics to be prepared (θ > 150° and sliding angle < 10°) [42,43,44]. Although this is considered to be an advantage of the dip-coating method, nevertheless, the use of nanoparticles is not preferred due to their ecological impact [45,46,47]. On the other hand, Qiang et al. [48] succeeded in preparing superhydrophobic cotton fabric without adding nano-particles via the dip-coating method and UV curing, using tri-functionality vinyl perfluoro decanol, vinyl-terminated polydimethylsiloxane and octavinyl-polyhedral oligomeric silsesquioxane as low surface energy agents with THF as a solvent. However, the use of fluorine compounds, especially C8 perfluoroalkyl chains, is not favorable due to their environmental impact and potential toxicity [49,50]. 

While the preparation of superhydrophobic fabrics typically requires the use of nanoparticles or fluorine compounds, it is also possible to create a highly hydrophobic fabric close to the superhydrophobic one without using nanoparticles or fluorine compounds. This alternative fabric can effectively serve various applications, including self-cleaning, all while maintaining the environmental safety.

Flax is an ideal and versatile choice for household textiles like bed linen and furnishing fabrics. It is also interesting for many applications such as kitchen fabrics, sails, tents and canvas. Moreover, short flax fibers can play a key role as reinforcement in composite materials, finding use in automotive interiors and furniture [51]. Thus, the preparation of hydrophobic flax fibers can be useful for several applications. 

In this study, polysiloxane-coated flax fabrics were prepared via the dip-coating method using (ethylhydrosiloxane-dimethylsiloxane) copolymer trimethylsiloxane terminated (PDMS-co-PHMS) with 1,3,5,7-tetravinyl-1,3,5,7-tetramethylcyclotetrasiloxane (D4V) as a curing agent and acetone as a solvent. The curing process was carried out via hydrosilylation with the Karstedt catalyst at room temperature. The coated fabrics displayed highly hydrophobic and durable properties with good self-cleaning ability for certain pollutants. Moreover, the effect of the coating on the mechanical properties of fabrics in terms of E modulus, tensile stress at maximum force (Fmax) and elongation at maximum force dL (Fmax) was evaluated. Furthermore, we observed that this effect can be controlled by adjusting the proportion of curing agent in the coating solution.

## 2. Results and Discussion

### 2.1. Effect of the Concentration of Coating Solution on the Coating Rate

**Figure 1:**
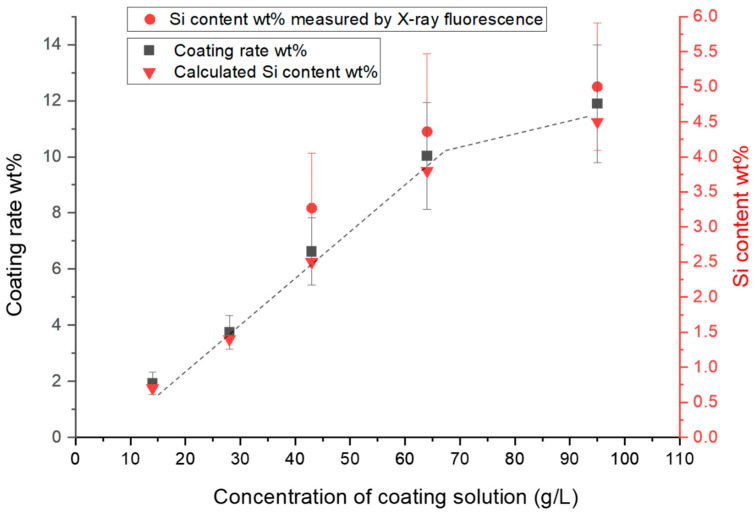
CR and calculated and measured Si content in the coated fabric (samples 01–05) as a function of the concentration of the coating solution.

As mentioned in Table 1, the fabric samples from 01 to 05 were coated using solutions of different silicone concentrations (from 14 to 95 g/L). The other parameters remained the same, even the Si-H/Si-vinyl ratio was 1/1 for all these samples. The coating rate (CR) of each coated sample was calculated using Equation (1) and the Si content of samples was calculated via Equation (2). Then, each treated sample was scanned with an X-ray fluorescence elemental analyzer in order to measure the Si content using Equation (5). However, samples 01 and 02 were not considered because they are outside of the linear domain used to the measure Si content. Indeed, the Si concentrations of these samples were estimated to be lower than 2 wt%. As expected, as the concentration of coating solution increased, the CR increased until it reached 95 g/L, where CR value began to stabilize at 12 wt%. On the other hand, the calculated amount of Si element in samples corresponded to the measured amount, which is implicitly consistent with CR, confirming its gradual increase with the increase in the concentration of the PDMS coating solution (Figure 1). 

These results show that the amount of coating needed for the treated fabric can be controlled by adjusting the concentration of the coating solution.

Fabric samples from 06 to 08 were coated with the same concentration of coating solution, but with different Si-vinyl/Si-H ratios. These samples exhibited identical CR (Table 2), confirming that changing the crosslinking agent ratio does not impact the amount of coating applied to the fabric.

**Figure 2:**
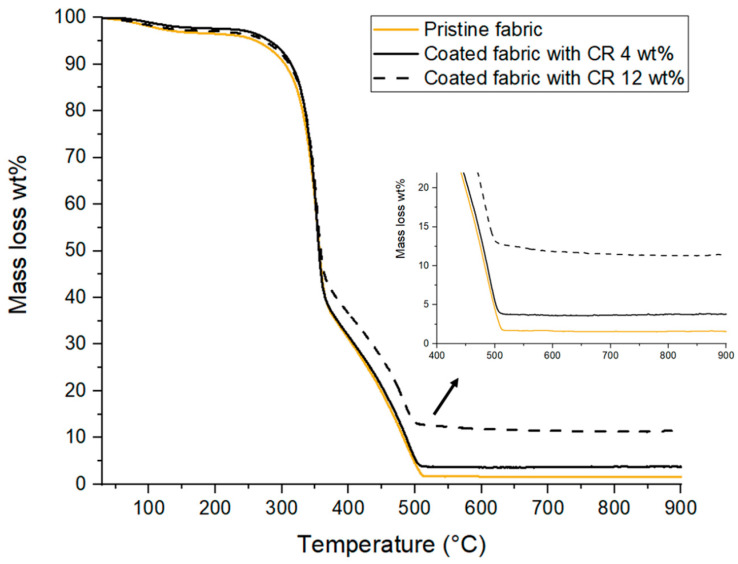
TGA curves of pristine and coated fabrics with 4 and 12 wt% of silicone.

TGA was performed on pristine and coated fabrics (samples 02 and 05 with CRs of 4 and 12 wt%, respectively) in order to follow the increase in residual values as well as the potential change to the thermal behavior of the fabrics due to the coating (Figure 2). 

The temperature at 5 wt% weight loss of pristine fabric is 260 °C, while for the coated fabrics with 4 and 12 wt% of silicone, it is almost the same value: 280 °C. Thus, there is a small delay in decomposition due to the PDMS coating. On the other hand, the highest rate of degradation of all samples occurs at 352 °C, without any difference. Finally, the residue remains stable between 500 °C and 900 °C with a clear difference in terms of final residue yield. The residual ash for samples increased from 1.6 to 3.7 and 11.4 wt% for pristine and for fabrics coated with 4 and 12 wt% of coating, respectively. This increase is consistent entirely with the residue obtained during the fire test from the silicone layer (Figures S1 and S2). 

### 2.2. Characterization of Silicone Coating

**Figure 3:**
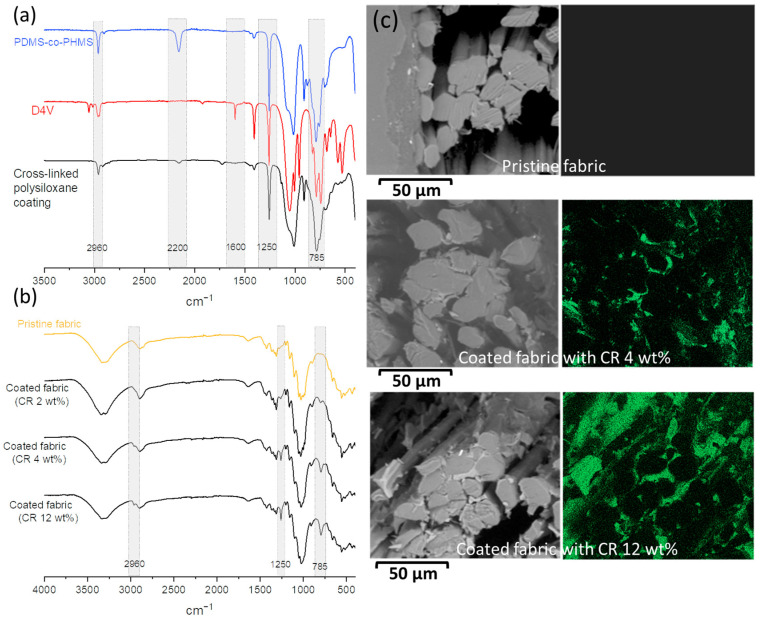
FTIR spectra for (**a**) PDMS-co-PHMS, D4V and the coating-based crosslinked polysiloxane. (**b**) Pristine and coated flax fabrics. (**c**) SEM/EDS images for pristine and coated fabrics of 4 and 12 wt% of CR.

PDMS-co-PHMS, D4V and the produced polysiloxane coating were analyzed by FTIR spectroscopy to confirm the hydrosilylation reaction and the formation of the cross-linked polysiloxane layer (Figure 3a). The bands corresponding to Si-H groups at 2200 cm^−1^ and Si-CH_3_ at 1250 cm^−1^, the C-H stretching of methyl groups at 2960 cm^−1^ and C–Si stretching at 785 cm^−1^ are observable in the PDMS-co-PHMS spectrum. On the other side, the bands corresponding to the vinyl groups C=C at 1600 cm^−1^, the Si-CH_3_ groups at 1250 cm^−1^, C-H stretching of methyl groups at 2960 cm^−1^ and C–Si stretching at 785 cm^−1^ are highlighted in the D4V spectrum. In the spectrum of produced coating, the bands corresponding to Si-CH_3_, C-H stretching of methyl and C–Si stretching are still observable, while the bands of Si-H and vinyl C=C groups have disappeared. This proves that the hydrosilylation reaction occurred between Si-H and vinyl groups of PDMS-co-PHMS and D4V, respectively. 

On the other hand, the pristine and the coated fabrics (01, 03 and 05) were analyzed using FTIR spectroscopy to confirm the success of the coating process (Figure 3a,b). The corresponding bands of coating film (Si-CH_3_, C-H stretching of methyl groups and C–Si stretching) are also observable in the spectra of coated fabrics but are not present for the pristine one. Also, the intensity of these bands increased with the increase in the CR of coated fabrics.

The fiber cross-sections of the pristine and coated fabrics were also analyzed by SEM/EDS to obtain silicon mapping and confirm the presence of the polysiloxane coating on the flax fibers’ surface. As is clearly confirmed in the SEM-EDS images (Figure 3c), silicon is not present in pristine fabric. However, in the case of coated fabrics, silicon becomes readily apparent in the fabric when CR is 4 wt%, equating to a silicon content of less than 2 wt%. Furthermore, the presence of silicon becomes more noticeable in the fabric with CR of about 12 wt%, corresponding to a silicon content of nearly 5 wt%.

It is worth noting that silicon is not present in the bulk of the fibers for the coated fabric and its presence is limited only to the elementary fiber surface, which plays the role of combining the fibers. This is particularly notable in the coated fabric with CR 12 wt%, which appears as dark areas in the EDS image. The penetration of silicon compounds inside the fiber is probably too difficult, probably due to their poor affinity with the materials which compose the flax fibers.

### 2.3. Hydrophobicity and Durability of Coated Fabrics

**Figure 4:**
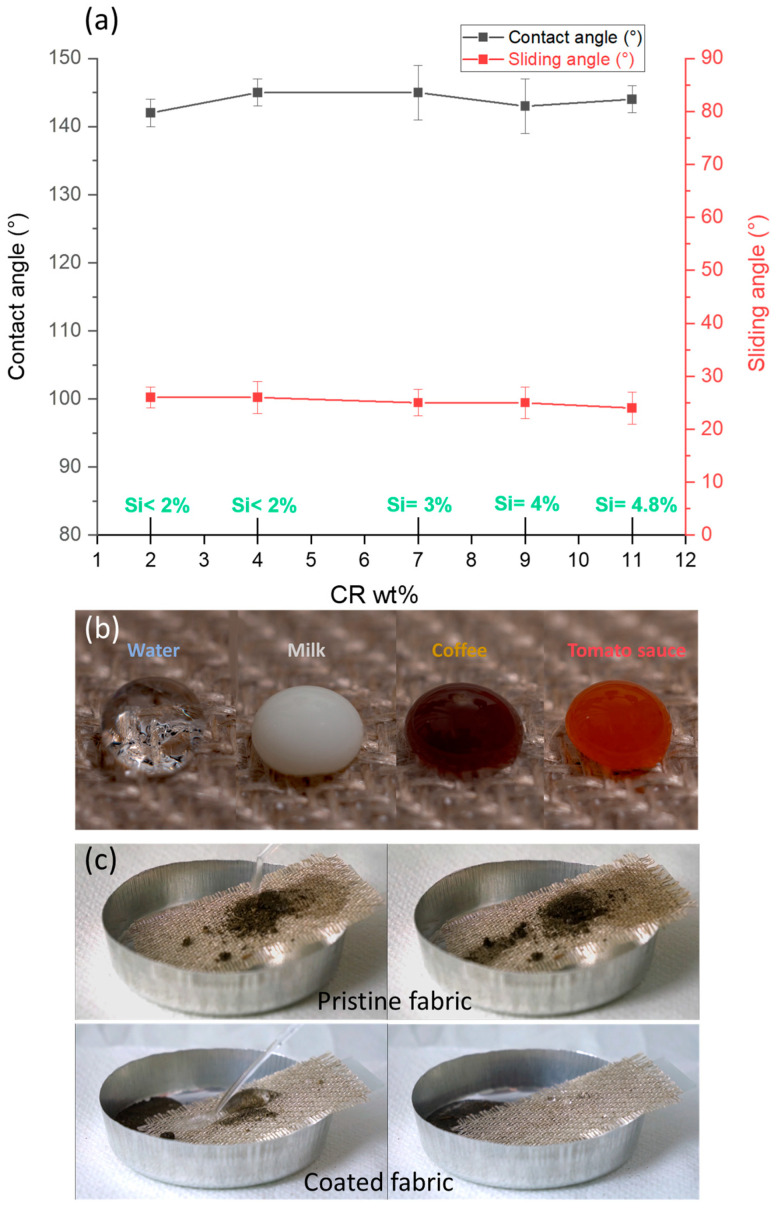
(**a**) Contact and sliding angles for coated samples with different silicone contents. (**b**) Images of drops of milk, coffee and tomato sauce deposited onto the surface of the coated fabric. (**c**) Self-cleaning performance of coated fabric for mud stains.

The fabric samples from 01 to 05 that have been coated using solutions of different concentrations contained different amounts of the silicon element. Nevertheless, they displayed similar hydrophobic properties. Contact and sliding angle measurements were performed on the coated fabrics to evaluate the hydrophobic properties. All samples had almost the same values: θ around 145° and SA around 23° (Figure 4a). This means that the amounts of silicon obtained for our samples were too high to show significant differences between them. The lowest amount of silicone obtained in this study, less than 2 wt% in our case, was sufficient to impart high hydrophobicity to flax fabric. Thus, increasing the silicon content beyond this limit is useless. 

When comparing our results to the research conducted by Taibi et al. [52], who successfully achieved a superhydrophobic flax fabric by grafting fluorinated (meth)acrylic monomers, we noticed a similar contact angle in both studies, approximately 145° in our research and around 150° in Taibi’s work. However, a notable difference appeared in terms of SA, with our fabric measuring about 23° while Taibi’s exhibited a significantly lower result, near to 9°. However, the preparation of a fluorine-free hydrophobic fabric, even with slightly reduced hydrophobic properties, offers versatility for a wide range of applications while upholding environmental safety.

On the other hand, contact and sliding angle measurements were also performed on samples 06 to 08 to evaluate the hydrophobic properties. All samples exhibited the same θ, around 145°, and SA near to 23° (Table 3), confirming that the crosslinking density of the coating has no effect on the hydrophobic properties of the fabrics.

The durability of hydrophobic coated fabrics has been investigated using a lab-simulation of home washing. X-ray fluorescence elemental analysis was used to follow the decrease in silicon content in the coated fabrics due to the removal of polysiloxane coating during the washing process. The durability test was performed on the coated sample (No. 05), which had an initial Si content of 5.2 wt%. There was no significant change in the X-ray fluorescence intensity of the Si element for this sample before and after four washing cycles (Figure S3). This confirms the good durability and fastness of the coating on the fabrics.

On the other hand, the coated fabric displayed a good self-cleaning performance. As shown in Figure 4b,c, drops of milk, coffee and tomato sauce were deposited onto the coated fabric. The coated fabric surface demonstrated a good repelling property, where the liquid drops formed a spherical shape on the surface.

Also, some mud was deposited onto the pristine and coated fabrics. The contaminants were easily taken away and rolled on the coated fabric by the moving water droplets, resulting in a completely clean surface, while the contaminants on pristine fabric were difficult to clean in a similar way; they left clear stains on the fabric surface after dropping water over it.

This indicates the efficacy of the coated fabrics on the self-cleaning property. These tests are inspired from References [8,48,53,54]. Two videos in The Supporting Information section allow you to visualize the water-repellent effectiveness of the coating.

### 2.4. Mechanical Properties of Coated Flax Fabrics

A tensile test was performed on the threads of pristine and coated fabrics in order to investigate the evolution of the mechanical behavior of flax fabric before and after the coating process. Also, the influence of the cross-linking density of the silicone coating on the mechanical properties of flax fibers was investigated. Indeed, the crosslinking density of silicone rubber had an effect on the mechanical properties [55]. As the D4V molecule plays the role of the cross-linking agent in our coating formulation, fabric samples 05 to 08 (Table 1) were coated using solutions with different [Si-vinyl/Si-H] ratios (from 1/1 to 1/4) by modifying the D4V content with the same other parameters. The coating rate of these samples was the same, while the difference was also the crosslinking density. Table 4 shows the results of (Fmax), dL(Fmax), F(rupture), dL(rupture) and E modulus for the pristine and coated samples with different Si-vinyl/Si-H ratios. 

**Figure 5:**
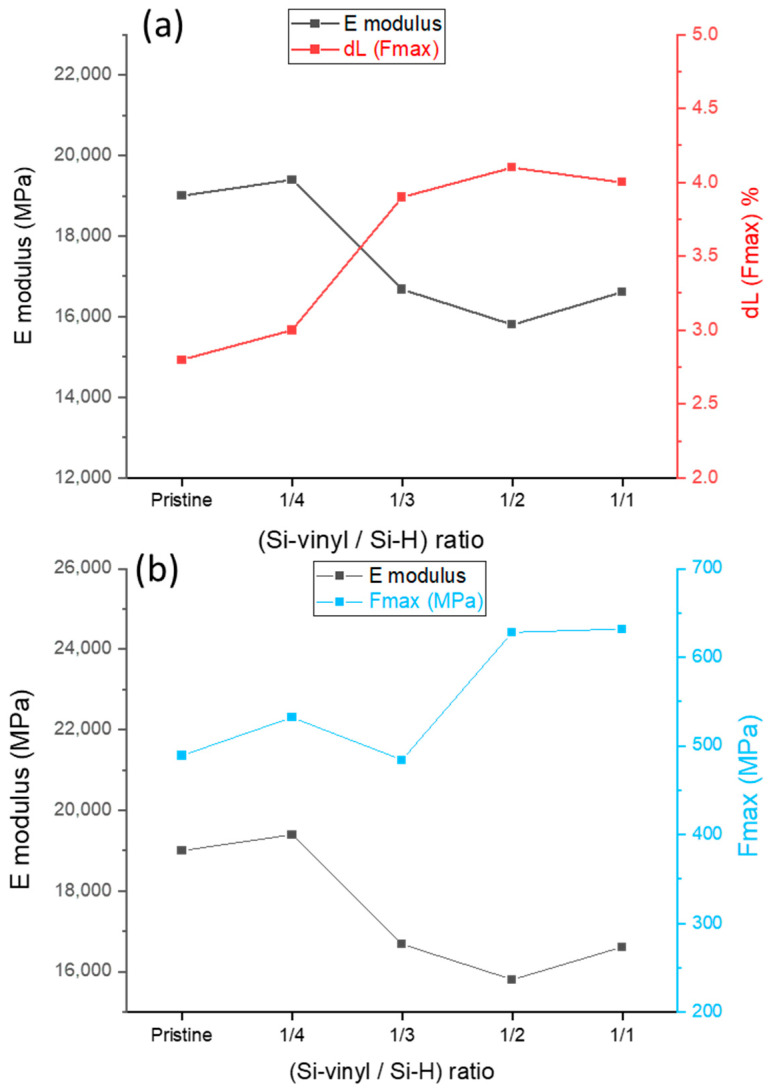
(**a**) Elongation and E modulus, (**b**) F(max) and E modulus for pristine and coated threads with different Si-H/Si-vinyl ratios.

The initial characteristics of pristine flax thread included an elongation at the Fmax value of approximately 3%, and an E modulus of 19,000 MPa. After coating with a Si-vinyl/Si-H ratio of 1/4, these values remained nearly unchanged. In contrast, when applying ratios of 1/3, 1/2 and 1/1, the elongation at Fmax increased to approximately 4%, while the E modulus decreased to nearly 16,000 MPa, representing changes of 33% and 15%, respectively. These results highlight the considerable impact of an elastomer polysiloxane coating on flax fabric, especially when the crosslinking density of the coating is high (Figure 5a). However, applying a polysiloxane coating with a ratio of 1/4 gives the fabric good hydrophobic properties without affecting the mechanical characteristics.

Furthermore, the Fmax for the pristine fabric and the fabric coated with 1/4 and 1/3 ratios was approximately 500 MPa. However, it increased to 600 MPa for ratios of 1/2 and 1/1, marking an increase of 20%. This increase confirms the necessity of increasing the crosslinking density of the coating in order to affect the mechanical properties (Figure 5b).

## 3. Materials and Methods

### 3.1. Materials

1,3,5,7-Tetravinyl-1,3,5,7-tetramethylcyclotetrasiloxane (97%), octamethylcyclotetrasiloxane (D4, 97%) and (25–35% ethylhydrosiloxane)–dimethylsiloxane co-polymer trimethylsiloxane terminated (25–35 cSt, Mn = 2000 g/mol) were purchased from ABCR. Platinum-divinyltetramethyldisiloxane complex (2% Pt) in xylene (Karstedt catalyst) and acetone (99%) were bought from Gelest. Liquid detergent containing 5–15% anionic surfactants, 5% non-anionic surfactants, amphoteric surfactants and enzymes was supplied from Enzpin. All these chemical products were used without further purification. Flax fabric was obtained from Hexcel (France). The composition of flax fibers was 81 wt% of cellulose, 13 wt% of hemicellulose and 2.7 wt% of lignin.

### 3.2. Preparation of Polysiloxane Coating Films

**Figure 6:**
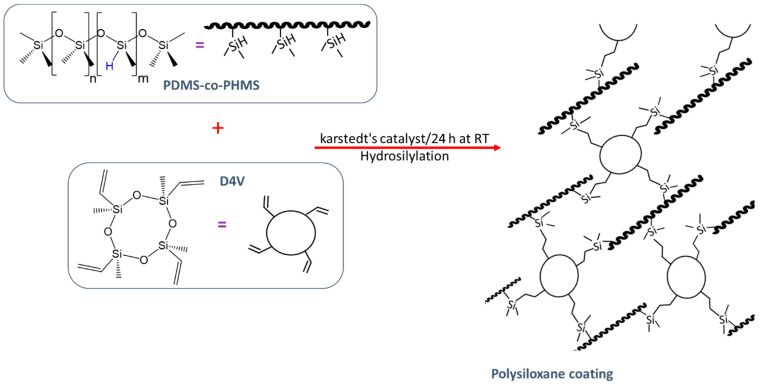
Schematic representation of the formation of the cross-linked polysiloxane coating.

In one beaker, 2.24 g of PDMS-co-PHMS with an equivalent quantity of D4V (0.81 g) was added to 32 mL of acetone and stirred for 10 min to achieve a homogeneous solution. Then, Karstedt catalyst (100 μL) was added followed by stirring for another 10 min. Afterwards, droplets of this solution were dropped onto a glass slide and left under a hood for 24 h until the termination of the hydrosilylation reaction and the formation of the crosslinked polysiloxane coating on the surface of the glass slide (Figure 6).

### 3.3. General Procedure for the Preparation of Hydrophobic Flax Fabrics via the Dip-Coating Method

**Figure 7:**
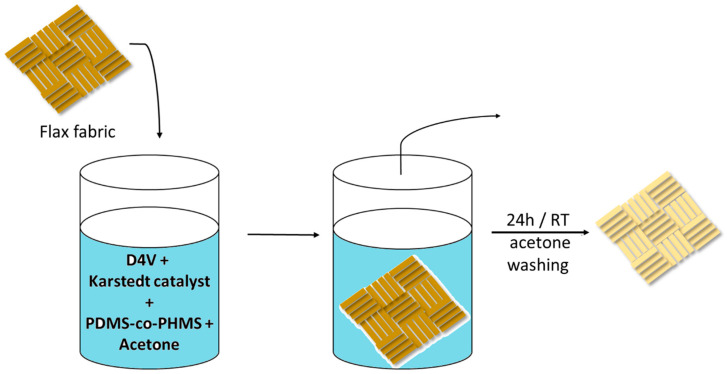
Scheme of the preparation of the hydrophobic flax fabrics via dip-coating method.

Briefly, 3 g of flax fabrics were cut into pieces with dimensions of 4 × 6 cm^2^. In one beaker, a given amount of PDMS-co-PHMS with an equivalent quantity of D4V was added to 32 mL of acetone and stirred for 10 min to achieve a homogeneous solution. Then, Karstedt catalyst was added, followed by stirring for another 10 min. The fabric samples were then immersed in the treatment solution for 5 min. Afterwards, the fabrics were removed and left under the hood for 24 h until a complete hydrosilylation reaction and the formation of coating-based crosslinked PDMS occured. Finally, the fabrics were washed with acetone to remove unreacted compounds and dried for 30 min at room temperature (Figure 7).

During the hydrosilylation reaction, cross-linking occurs between the PDMS-co-PHMS and D4V, producing the polysiloxane layer that coats and traps the flax fibers, ensuring better adhesion of the coating to fabrics.

Conditions for the preparation of the different fabric samples are summarized in Table 1. 

The coating rate (CR wt%) was calculated according to the percentage ratio between the weight of the coated fabric and that of the corresponding pristine one.

(1) $$\mathrm{CR}\;\mathrm{wt}\%=\left(\frac{\mathrm{Coated}\;\mathrm{fabric}\;\mathrm{weight}\;-\;\mathrm{pristine}\;\mathrm{fabric}\;\mathrm{weight}}{\mathrm{Coated}\;\mathrm{fabric}\;\mathrm{weight}}\right)\times 100$$

The calculated Si content wt% can be estimated considering that polysiloxane coating consists of –[O-Si(CH_3_)_2_]- unit, as follows:

(2) $$\mathrm{Calculated}\;\mathrm{Si}\;\mathrm{content}\;\mathrm{wt}\%=\left(\frac{\mathrm{CR}\;\mathrm{wt}\%\times 28}{74}\right)$$

where 28 and 74 are the molecular weights of the Si and –[O-Si(CH_3_)_2_]- group, respectively.

### 3.4. Characterizations

#### 3.4.1. Fourier Transform Infrared Spectroscopy (FTIR) 

Fourier transform infrared spectra of fabrics were obtained using a Bruker VERTEX 70 spectrometer used in attenuated total reflectance mode, by performing 32 scans between 400 and 4000 cm^−1^.

#### 3.4.2. Scanning Electron Microscope Coupled with Energy Dispersive X-ray Spectrometer SEM-EDS

To locate the presence of the silicon element from the coating, the coated flax fabrics were analyzed using a scanning electron microscope (FEI Quanta 200) coupled with an energy dispersive X-ray spectroscopy, EDS (Oxford INCA Energy system, Oxford Instruments, UK).

After being cut with a single edge blade, the samples were placed on a vertical sample holder under a high vacuum at a voltage of 12.5 kV and a working distance of 10 mm. 

#### 3.4.3. X-ray Fluorescence Elemental Analysis

Silicon content in the coated flax fabrics was determined using Oxford XMET 5100 X-ray fluorescence. Samples were fixed on a flat polymer-based support containing no trace of silicon. This holder is used to flatten fabric samples in order to reduce measurement errors. The analyses were carried out under atmospheric pressure, without any preparation. The following settings were used by default: 13 kV and 45 μA. Each spectrum was collected for 1 min. The intensity of the silicon peak is proportional to its concentration in the fabric sample. The measurement was carried out 3 times for each sample.

To obtain the calibration curve, a series of octamethylcyclotetrasiloxane (D4) solutions with different concentrations was prepared using acetone as a solvent. Then, each solution was used to coat a flax fabric sample (2 × 2 cm^2^) by immersing for 3 min. Finally, the flax fabric samples were dried for 1 h under the hood. These samples, impregnated with D4, were defined as the standard series. The weight gain of each sample of this standard series was calculated using Equation (2):

(3) $$\mathrm{Weight}\;\mathrm{gain}\;\mathrm{of}\;D4\left(\%\right)=\frac{\left(\mathrm{Sample}\;\mathrm{initial}\;\mathrm{weight}\right)\;-\;\left(\mathrm{Sample}\;\mathrm{final}\;\mathrm{weight}\right)}{\left(\mathrm{Sample}\;\mathrm{final}\;\mathrm{weight}\right)}\;\times \;100$$

Then, the value of the weight gain of each sample was used to calculate the silicon content using Equation (3):

(4) $$\mathrm{Si}\;\mathrm{content}\;(\mathrm{wt}\%)=\;\frac{\left(\mathrm{Weight}\;\mathrm{gain}\;\mathrm{of}\;D4\;\%\right)\;\times \;(4)\;\times \left(\mathrm{Molar}\;\mathrm{mass}\;\mathrm{of}\;\mathrm{Si}\right)}{\left(\mathrm{Molar}\;\mathrm{mass}\;\mathrm{of}\;D4\right)}$$

where (4) is the number of Si atom in D4.

The standard samples were then scanned using an X-ray fluorescence spectrometer (Figure S4). The values of fluorescence peak areas of the silicon element for the standard samples were drawn as a function of silicon content in order to obtain a calibration curve (Equation (4)). The equation of the resulting linear curve was then useful to determine the silicon content of unknown samples from direct X-ray fluorescence spectrometry analysis.

(5) $$X=\;\frac{Y+20.8}{32.8}$$

where X is the silicon content (wt%) and Y is the area value of the silicon fluorescence peak.

#### 3.4.4. Contact and Sliding Angles’ Measurement

Krüss DSA 30 apparatus was used to measure the contact angle (θ) of water with treated fabrics. Measurements were carried out 3 times at 5 different places on the coated fabric sample with a water drop volume of 3 μL. The sliding angle (SA) was measured using a lab-made device. The device is a vertical metal axis crossed with a manually movable horizontal one which holds a protractor on the side and a sample carrier above. The coated fabric is placed on the sample carrier and a drop of water (50 μL) is put on the fabric surface. By moving the horizontal axis, the water drop rolls over the fabric surface. The SA is the angle at which the water drop starts to move out of its initial position. 

#### 3.4.5. Thermogravimetric Analysis (TGA)

A thermogravimetric analyzer instrument (Setaram Setsys) was used to study the thermo-oxidative decomposition of the treated fabrics. This analysis allows the coating amount in the sample to be evaluated via the increase in residues at the end of the test. In alumina ceramic crucibles, 10 mg ± 1 mg of the fabric sample was heated from 30 to 900 °C (heating rate of 10 °C/min) under an air atmosphere (40 mL/min). During the analysis, 15 min of isotherm at 100 °C was carried out in order to remove adsorbed water and facilitate the comparison between samples.

#### 3.4.6. Durability Test

Four samples of coated fabric (0.6 g) were washed in 100 mL of detergent solution (4 g/L) at 40 °C for 10 min under stirring at (200 rpm). This test can be considered to be a lab-simulation of home washing [56].

Then, the solution was removed and the fabric underwent several washes with deionized water to remove all traces of the detergent solution. The wash cycle was repeated 4 times. X-ray fluorescence elemental analysis was used to detect the loss of the silicon element after the wash cycles. 

#### 3.4.7. Tensile Test

The tensile test of the fabric thread was performed on a tensile machine (Zwick/Roell) equipped with a 2.5 kN load cell and a clip-on extensometer with a reference length of 30 mm at room temperature (25 °C).

A crosshead speed of 10 mm/min was applied with a pre-charge of 1 N. The Young’s modulus was determined by a tangent method between 0.5 and 2% of elongation. Each test was carried out at least 4 times.

Considering the threads as a cylinder, their diameter averages were calculated as below:

(6) $$D=\;\sqrt{\frac{4\;\times \;\left(\frac{m}{\rho }\right)}{L\;\times \;\pi }}$$

where m is the weight of the thread, *ρ* is the density and L is the length.

This test is adapted from Ferrara’s study [57], in which a tensile strength test of flax fabrics was performed. 

#### 3.4.8. Density Measurement

The density of the thread samples was measured using a micromeritics AccuPyc 1330 gas pycnometer. The sample was enclosed in an instrument compartment of known volume, and the inert gas (Ar) was introduced and then expanded into another precision internal volume. The pressure before and after expansion was measured and used to calculate the sample volume. Dividing the sample weight into this volume gives the sample density. The measure was repeated at least 3 times for 5 samples of each thread.

#### 3.4.9. Preliminary Fire Test on Fabrics

A non-standardized fire test was performed to evaluate the flammability of coated fabric samples and calculate the char residue [58]. These fabric samples, approximately 0.5 g and measuring 5 × 2 cm^2^, were fixed between two glass plates and positioned vertically. To ignite the upper surface of the fabrics, a cigarette lighter was employed. This method serves to assess the fabrics’ ability to self-extinguish and their capacity to prevent the spread of flames. After complete combustion, the remaining residue was weighed. To determine the percentage of mass loss, the weight of the fabric section pinched between the plates was subtracted from both the initial weight and the residue weight. These values can be consistent with those obtained by TGA test. The details of the test are illustrated in the supplementary video from our previous work [58].

## 4. Conclusions

In this study, flax fabrics were successfully coated with polysiloxane via a hydrosilylation reaction in the presence of the Karstedt catalyst. The quantity of silicone coating applied to the fabric’s surface was controlled by adjusting the concentration of the coating solution. These coated fabrics exhibited excellent hydrophobic properties with good self-cleaning capabilities. Moreover, they displayed exceptional durability over four washing cycles. Interestingly, the amount of coating on the fabric’s surface has no effect on its hydrophobic characteristics. Even a small quantity of coating, representing approximately 2 wt% of Si, is enough to confer hydrophobicity upon the fabrics. The polysiloxane coating impacts the mechanical properties of the flax fabric, but this impact can be controlled by adjusting the crosslinking density of the coating, without affecting the fabric’s hydrophobic nature. 

## Figures and Tables

**Table 1:** Conditions for the preparation of the different flax fabric samples using coating solutions of different PDMS concentrations and Si-H/Si-vinyl ratios.

Sample No.	Flax Fabrics (g)	PDMS-co-PHMS (g)	D4V (g)	Acetone (mL)	Karstedt Catalyst (μL)	Concentration (g/L)	Si-Vinyl/Si-H Ratio
01	3	0.34	0.12	32	15	14	1/1
02	3	0.67	0.24	32	30	28	1/1
03	3	1.00	0.36	32	45	43	1/1
04	3	1.50	0.54	32	67	64	1/1
05	3	2.24	0.81	32	100	95	1/1
06	3	2.58	0.46	32	50	95	1/2
07	3	2.72	0.33	32	33	95	1/3
08	3	2.80	0.25	32	25	95	1/4

**Table 2:** Coating rate for the coated fabric with different Si-vinyl/Si-H ratios.

Sample No.	Si-Vinyl/Si-H Ratio	Coating Rate wt%
6	1/2	11.4 ± 1
7	1/3	10.7 ± 2
8	1/4	10.8 ± 1

**Table 3:** Contact and sliding angle values for the coated fabric with different Si-vinyl/Si-H ratios.

Sample No.	Si-Vinyl/Si-H Ratio	θ (°)	SA (°)
6	1/2	147 ± 4	23 ± 3
7	1/3	145 ± 3	23 ± 2
8	1/4	144 ± 5	22 ± 4

**Table 4:** Results of tensile test on the threads of pristine and coated fabrics with different Si-vinyl/Si-H ratios of polysiloxane coating.

Sample No.	Si-Vinyl/Si-H Ratio	CR (wt%)	Fmax (Mpa)	dL (Fmax) %	Frupt (MPa)	dL (Rupture) %	D (mm)	Module E (MPa)
Pristine	-	-	489 ± 61	2.7 ± 0.3	413 ± 190	2.8 ± 0.3	0.27	19,000 ± 1140
5	1/1	11.6 ± 3	632 ± 112	4 ± 0.4	609 ± 120	4 ± 0.5	0.27	16,612 ± 1900
6	1/2	11.4 ± 1	628 ± 92	4.1 ± 0.6	593 ± 74	4.2 ± 0.6	0.27	15,801 ± 2245
7	1/3	10.7 ± 2	484 ± 27	3.9 ± 1.5	450 ± 79	4.1 ± 1.6	0.27	16,682 ± 700
8	1/4	10.8 ± 1	532 ± 89	3 ± 0.3	451 ± 147	3.2 ± 0.2	0.27	19,398 ± 2140

## Data Availability

Data are contained within the article and Supplementary Materials.

## Supplementary Materials

The following supporting information can be downloaded at: https://www.mdpi.com/article/10.3390/molecules29040829/s1, Figure S1: Fire test for pristine fabric and coated fabric with Si content (5 wt%). Figure S2: The residue obtained by fire test and TGA test as a function of the Si content. Figure S3: X-ray fluorescence intensity of Si element for the coated sample (N° 05) before and after 4 washing cycles. Figure S4: X-ray fluorescence peak area as function of silicon content of the known flax fabric samples with the drawn linear equation.
