# Supplementary material for: A One-Step Approach for a Durable and Highly Hydrophobic Coating for Flax Fabrics for Self-Cleaning Application
Source: Molecules. 2024 Feb 13;29(4):829. doi: 10.3390/molecules29040829 (PMC10891639; doi:10.3390/molecules29040829)
Supplement: Supplementary file 1 [file molecules-29-00829-s001.zip › molecules-2814246-supplementary.pdf]

One-step approach to durable and highly hydrophobic coating for flax fabrics for self-cleaning application.

Antoine Ishak, Rodolphe Sonnier, Belkacem Otazaghine * and Claire Longuet

PCH, IMT–Mines Alès, 6, Avenue de Clavières, 30100 Alès, France; antoine.ishak@mines-ales.fr (A.I.);
rodolphe.sonnier@mines-ales.fr (R.S.); claire.longuet@mines-ales.fr (C.L.)
* Correspondence: belkacem.otazaghine@mines-ales.fr

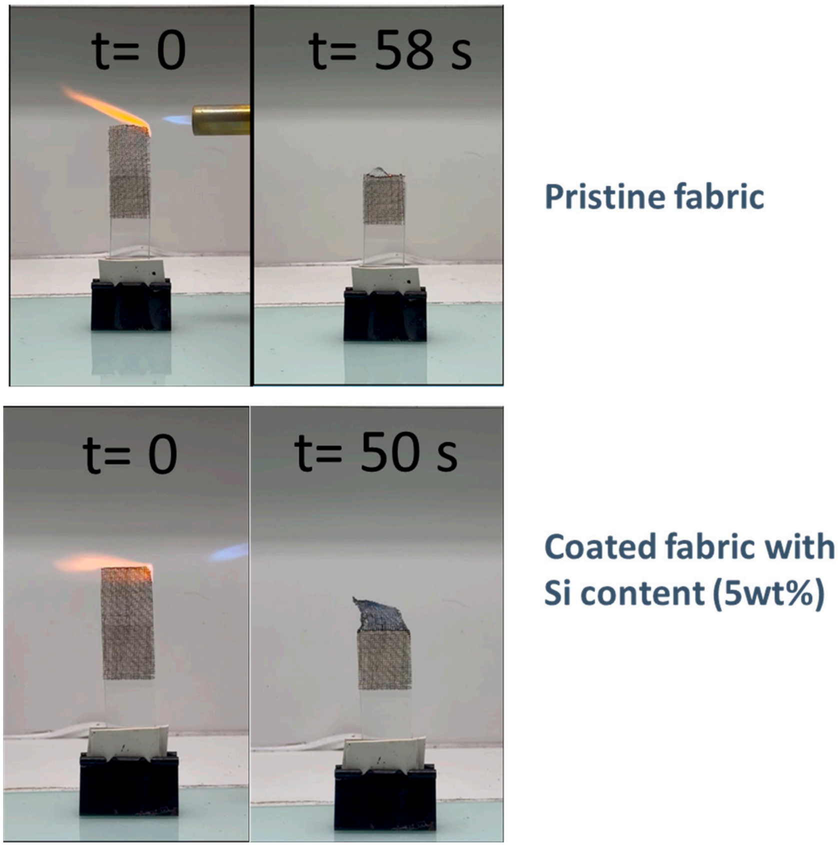

Figure S1: Fire test for pristine fabric and coated fabric with Si content (5 wt%).

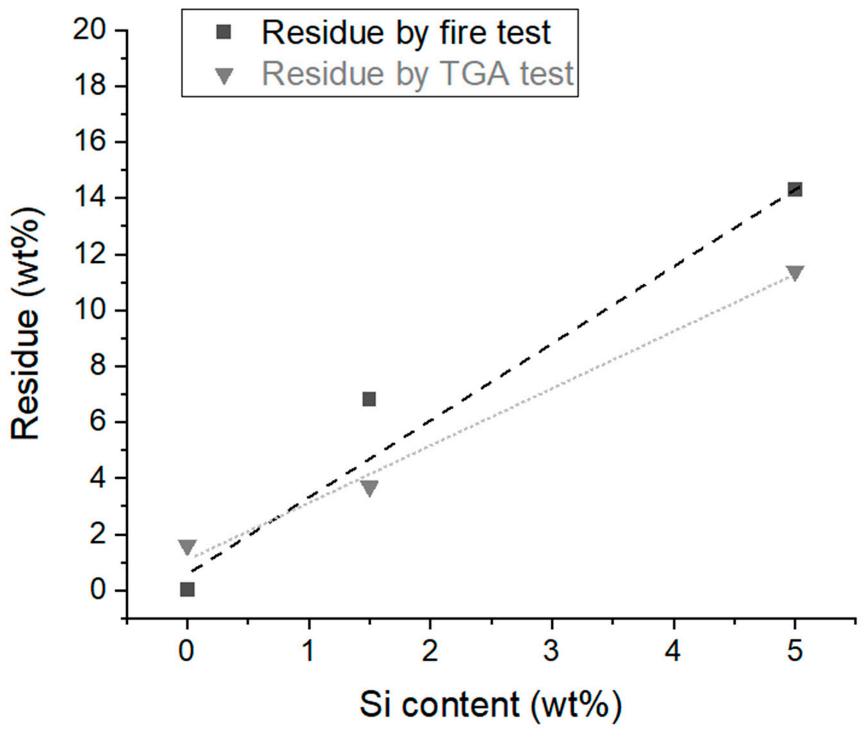

Figure S2: The residue obtained by fire test and TGA test as a function of the Si content.

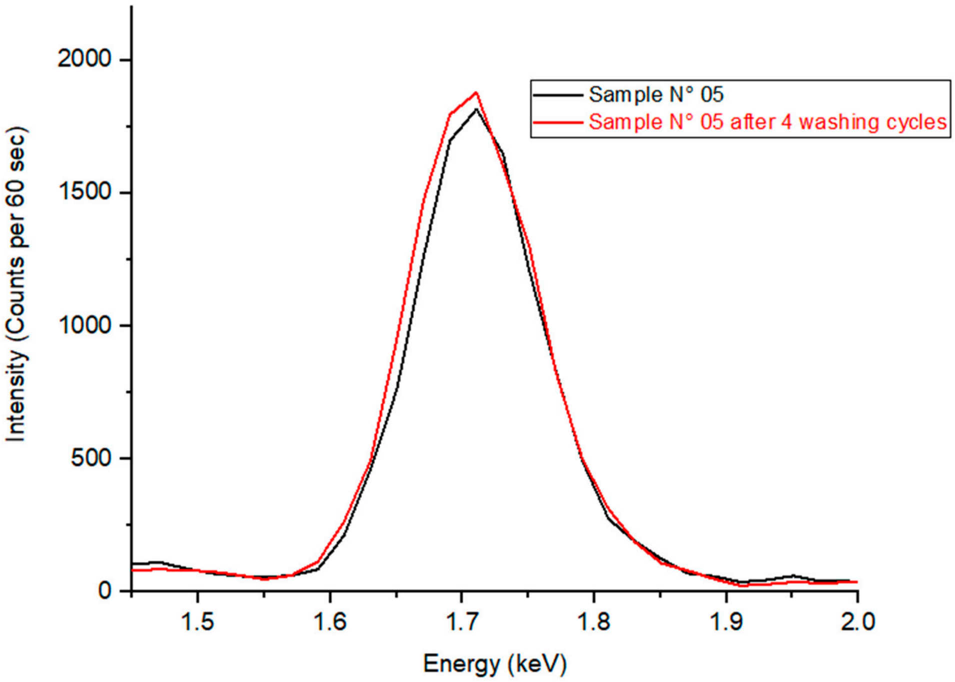

Figure S3: X-ray fluorescence intensity of Si element for the coated sample (N° 05) before and after 4 washing cycles.

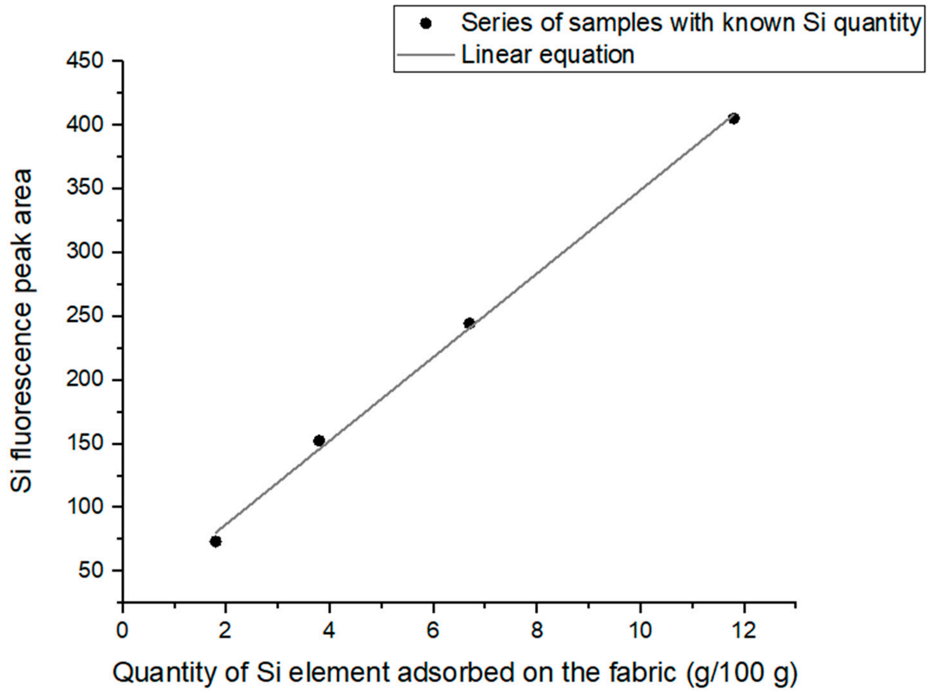

Figure S4: X-ray fluorescence peak area as function of silicon content of the known flax fabric samples with the drawn linear equation.
